# Negative workplace gossip and turnover intention among kindergarten teachers: psychological safety as a mediator and organizational identification as a moderator

**DOI:** 10.3389/fpsyg.2025.1588482

**Published:** 2025-06-18

**Authors:** Baoan Feng, Gaojie Dou, Xiaoqian Zhan

**Affiliations:** ^1^College of Teacher Education, Quzhou University, Quzhou, Zhejiang, China; ^2^College of Business, Quzhou University, Quzhou, Zhejiang, China; ^3^The Faculty of Art and Design, Quzhou College of Technology, Quzhou, Zhejiang, China

**Keywords:** psychological safety, organizational identification, negative workplace gossip, turnover intention, kindergarten teachers

## Abstract

**Purpose:**

Empirical evidence examining factors influencing turnover intention among early childhood educators remains scarce, especially research on negative workplace gossip as the influencing factor. To address these shortcomings, this study empirically analyzed whether and how negative workplace gossip, psychological safety, and organizational identification affect kindergarten teachers' turnover intention based on the Chinese cultural background.

**Methods:**

A cross-sectional research design was adopted. Self-reported questionnaire data were randomly collected from 1,872 Chinese kindergarten teachers through an online platform. The questionnaire included perceived negative workplace gossip scale, psychological safety scale, organizational identification scale, intent to leave scale, and demographic variables. A moderated mediation model linking negative workplace gossip, psychological safety, organizational identification, and turnover intention was tested using these data.

**Results:**

The study found that there was an association between negative workplace gossip and the turnover intention of kindergarten teachers. Psychological safety played a mediating role in this relationship, that is, negative workplace gossip influenced teachers' turnover intention by reducing their psychological safety. Meanwhile, organizational identification moderated the relationship between psychological safety and turnover intention. A higher level of organizational identification could weaken the negative impact of psychological safety on turnover intention.

**Conclusions:**

Kindergarten principals should take various measures to prevent the disorderly development of negative workplace gossip and enhance the psychological safety and organizational identification of kindergarten teachers, which are crucial for reducing the turnover intention of kindergarten teachers.

## Introduction

Employee turnover refers to individual employees actively or passively stopping or terminating their employment relationship with the organization or team (Akasheh et al., 2024). Early childhood educators exhibit some of the most elevated attrition rates across educational occupations (Schaack et al., 2020), and such high turnover rates are very common in many industrialized countries and form a serious worldwide problem (Kanchana and Jayathilaka, 2023; Wesonga and Van Der Westhuizen, 2024). The annual teacher turnover rates in childcare institutions in some Australian states are as high as 60%. Meanwhile, 51% of surveyed early childhood educators reported they would leave their jobs at any time if allowed (Jovanovic, 2013). Feng et al. (2017) found that one-third of early childhood educators had departed their positions in some Beijing kindergartens, while Totenhagen et al. (2016) pointed out that the annual national turnover rates of early childhood educators range from 24% to 40% in the United States. Similar situations also exist in Sweden (Matsuo et al., 2021); a survey in Sweden found that 48% of child welfare service practitioners had disclosed their intention to resign (Tayama et al., 2018).

Employee turnover in any profession has its pros and cons. Low employee turnover leads to organizational stagnation, while effective organizations usually promote innovation by eliminating low-level employees and bringing in “new blood” (Ingersoll et al., 2014). Many studies have, however, confirmed that the high turnover rates of kindergarten teachers can have many negative effects; teacher replacement can damage the closeness of the emotional connection between teachers and children, leading to anxiety in children (Herman et al., 2024; Howes and Smith, 1995), creating obstacles to the formation of intimate relationships between children and teachers (Cassidy et al., 2011). The turnover of preschool teachers may have a negative impact on young children's language and behavioral development (Markowitz, 2024), and even inducing to problematic behavior in children (Steimle and Ryan, 2023). Frequent occupational exits among teaching professionals could undermine the operational stability of kindergarten staff ecosystems (Whitebook et al., 2014) and increase the economic cost of recruiting and training new teachers (Wesonga and Van Der Westhuizen, 2024). As is well-known, the stability of the kindergarten teacher workforce is a key foundation for ensuring high-quality early childhood education and care services (Fenech et al., 2022). Given the systemic repercussions of unstable teaching teams, mproving kindergarten teachers' retention has emerged as a critical challenge in teacher management (Wesonga and Van Der Westhuizen, 2024).

Turnover intention most strongly precedes employees' resignation behaviors across organizational study (Ki and Choi-Kwon, 2022). Turnover intention refers to the consciously formed and purposeful inclination to depart from the organization (Fiset et al., 2017; Ngo-Henha, 2017). Previous studies have delved into the elements influencing employees' turnover intention, including job-related characteristics such as low salary (Matsuo and Higashijima, 2023; Schaack et al., 2020; Thorpe et al., 2020), poor benefits (Hur et al., 2023), high workload (Räsänen et al., 2020), job stress (Choi and Kim, 2020; Labrague, 2020), negative workplace gossip (He and Wei, 2022), difficult human relations (Matsuo and Higashijima, 2023; Moemi et al., 2023), as well as the attributes of individual teachers, including burnout (Charzyńska et al., 2021), psychological safety and psychological capital (Brohi et al., 2018; Gan and Cheng, 2021), organizational identification (Başar and Sigri, 2015), and subjective vitality (Collie, 2023). The aforementioned research findings systematically reveal the driving factors of employees' turnover intention from multiple perspectives, providing empirical evidence for in-depth analysis of the influencing mechanisms behind employees' turnover intention.

As a prominent factor exerting an impact on employees' turnover intention, negative workplace gossip has become a focus of academic attention in recent years. However, most of these studies focus on the corporate sector, and research on the group of kindergarten teachers is extremely scarce (He and Wei, 2022). Kindergarten teachers are certified early childhood educators who deliver structured pedagogy and care to children aged 3–6 years (varies by national frameworks) within formal early childhood education programs. Throughout the globe, women represent the overwhelming majority among kindergarten teachers (with a gender proportion exceeding 90%; Hong et al., 2022; Hsu et al., 2019; Petrić et al., 2022; Piperac et al., 2024), and their social interactions in the workplace are often associated with the social stereotype of “gossiping tendency” (Robbins and Karan, 2020). Some studies have indicated that women exhibit comparable levels of aggression to men, and women often employ relational aggression as their primary tactic, targeting predominantly female peers (Crothers et al., 2009; Namie, 2024). Additionally, given that the work of kindergarten teachers demands substantial emotional commitment and intensive energy expenditure (Sottimano et al., 2017), negative workplace gossip may not only reduce teachers' work engagement and strengthen their turnover intention, but may also undermine the quality of early childhood education and impede the healthy development of children (Wu et al., 2018; Yang et al., 2024). A study clearly demonstrates that uncivil behavior toward preschool teachers causes them significant harm, including stress, distress, anger, and other negative emotions, ultimately depleting their emotional resources (Itzkovich and Dolev, 2021b). Therefore, against the backdrop of Chinese cultural traditions, this study delves into the intricate pathways by which negative workplace gossip exerts its influence on the turnover intention of kindergarten teachers.

The impact of negative workplace gossip on turnover intention may be realized through the mediating role of psychological safety. Most existing literature has explored the mechanisms through which workplace incivilities (e.g., mistreatment, bullying, and deviant behaviors) influence employee turnover intentions (Dapilah and Druye, 2024; Faheem et al., 2023; Park, 2023). The workplace incivility is prevalent and has destructive effects on both employees and organizations (Itzkovich and Dolev, 2021a; Konuk and Koçak, 2025). These effects may trigger emotional, perceptual, and behavioral responses, such as increased anxiety, depleted emotional resources, and increased job insecurity (Irwin et al., 2025; Itzkovich and Dolev, 2021b), which in turn undermines the organizational climate and diminishes employees' psychological safety (Felblinger, 2008). A study on revenge behaviors among preschool teachers indicated that when preschool teachers are harassed by workplace incivility, it can trigger irritation and revenge behaviors (Itzkovich, 2024). The continuous accumulation of such negative emotions may gradually transform into turnover intention and intensify over time (Jasiński and Derbis, 2022). Although existing literature has extensively explored the impact of workplace incivility on employees' psychological safety and turnover intention, few studies have deeply focused on negative workplace gossip as a specific manifestation. As a typical manifestation of workplace incivility, negative workplace gossip is marked by more pronounced interpersonal targeting and information dissemination traits (Zou et al., 2020). By fostering an atmosphere of distrust and tension, it may directly undermine the psychological safety of gossip recipients and heighten their turnover intentions. In light of this, this study aims to transcend traditional research frameworks by focusing on negative workplace gossip and rigorously examining the mediating role of psychological safety in the relationship between such gossip and kindergarten teachers' turnover intentions. The goal is to offer novel theoretical and empirical contributions to understanding how workplace interpersonal dynamics shape employees' psychological and behavioral outcomes.

Additionally, while existing research has revealed the influence mechanism of employees' psychological safety on turnover intention (Liu and Keller, 2021), it has generally overlooked the moderating effect of organizational-level contextual factors. As a core psychological bond through which employees integrate organizational values into their self-concept (Ashforth and Mael, 1989), the level of organizational identification may alter the strength of psychological safety's impact on turnover intention through cognitive restructuring (e.g., perceiving gossip as an isolated incident), emotional buffering (e.g., a sense of belonging mitigating psychological threats), and behavioral consistency motivation (e.g., maintaining organizational image). However, the current literature has not tested this moderated mediating mechanism, particularly in emotion-intensive early childhood education settings, where whether organizational identification weakens or strengthens the mediating path of psychological safety remains empirically unclear. This study aims to fill this theoretical gap.

### Theoretical background and hypotheses

This study employed the bystander effect (Latané and Darley, 1968) as a framework to analyze how negative workplace gossip influences kindergarten teachers' turnover intention. When an incident requiring intervention occurs, bystanders are less likely to take action if they perceive the presence of others at the scene, which is termed the bystander effect (Darley and Latané, 1968; Latané and Darley, 1968). This study takes the bystander effect as the theoretical cornerstone, combines the two-way dissemination characteristics of negative workplace gossip (Noon and Delbridge, 1993), and constructs a “victim-bystander” dual-role interaction model to analyze the unique psychological mechanism of kindergarten teachers in negative workplace gossip. The bystander effect reveals the interactivity of individual behaviors within a group: when negative workplace gossip occurs, an individual may either be the target of the gossip (the victim) or become the recipient of the gossip information due to being involved in the dissemination chain (the bystander). According to the theory of dispersion of responsibility by Darley and Latané (1968), when gossip is actively informed by a third party, the “benevolent intervention” of the informant may alleviate the victim's sense of isolation (that is, “the positive behavior of the bystander weakens the transmission of pressure”); Conversely, when victims directly overhear negative gossip, bystander inaction exacerbates perceived ostracism, further undermining psychological safety (Edmondson, 2019) and increasing turnover risk.

### Negative workplace gossip and turnover intention

In the context of organizational behavior research, negative workplace gossip is defined as informal, evaluative communication among organizational members that focuses on criticizing an absent third party in a derogatory manner (Abdul et al., 2022; Kakarika et al., 2024). Negative workplace gossip (e.g., divorce, physiological defect, affairs, and the stigmatization of the target) is notorious for indirectly attacking and causing harm to the target (Beersma and Van Kleef, 2012), and is seen as a morally suspect activity (Eckhaus and Ben-Hador, 2018). This intervention could disrupt targets' emotional regulation and impair cognitive processing (Tian et al., 2018), privacy disclosure (Foster, 2004), reputation damage (Lee and Barnes, 2021), ego depletion (Ullah et al., 2021) and emotional exhaustion (Liu et al., 2020; Xie et al., 2019). These negative impacts may further induce withdrawal behaviors—including such as social withdrawal (e.g., avoiding teamwork, reduce the willingness to disclose information; Janatolmakan et al., 2025; Kawakatsu et al., 2024; Wellock, 2010), psychological withdrawal (e.g., slacking off and reducing work engagement; Dedahanov et al., 2025), and even physiological withdrawal (e.g., resignation and job transfer; Akerstrom et al., 2021; Esmaeilbeigi et al., 2025; Shatnawi et al., 2024). Negative workplace gossip heightens psychological pressure on targets (Cheng et al., 2022), thereby fostering withdrawal behavior as a means to evade hostile work environments (Zhao et al., 2021). For instance, if individuals are the targets of bullying for a long time, the development of their professional identity will face obstacles (Janatolmakan et al., 2025), or they even will have the intention to transfer from their current positions or leave the industry (Piri et al., 2024). So, the first hypothesis is formulated:

*Hypothesis 1: There exists a direct and positive relationship between negative workplace gossip and turnover intention*.

### Psychological safety as a mediating factor

Psychological safety refers to the shared belief among employees that they can speak up, take risks, and interact openly at work without fear of negative consequences (Edmondson, 1999). Psychological safety is not only reflected in the trust between people, but also includes the mutual respect between team members and can speak freely (Walumbwa and Schaubroeck, 2009). When individuals are more satisfied with their psychological safety within the organization, they can freely focus on the organization's goals rather than on self-protection (Edmondson and Lei, 2014). Employees suffering from negative workplace gossip—and thus a lack of psychological safety—may face great psychological pressure and have bad emotional experiences (e.g., anxiety, disappointment, anger, and depression; Michelson and Mouly, 2000). A study had shown a positive correlation between employees' perception of workplace incivility and their level of job insecurity (Itzkovich, 2016). The targets of negative workplace gossip suffer greater psychological pressure (Cheng et al., 2022), while a negative psychological climate at work can lead to emotional fatigue (Rentzou, 2012) or even trigger the deterioration of turnover intention (Jeon and Wells, 2018). After the development of psychological safety, however, teachers show positive behaviors related to morality, investment, and commitment, which increases the likelihood of reducing their turnover intention (Brohi et al., 2018).

This phenomenon can be interpreted through multiple theoretical frameworks. Social exchange theory contends that social exchange is a tangible or intangible social interaction process that takes place between at least two individuals (or groups) and is based on voluntariness, reciprocity, and trust. This process constitutes a social interaction mechanism of core significance in social life and is the core foundation of the relationships between individuals and groups (Blau, 1964). Negative workplace gossip, as an instance embodying negative reciprocity in social exchange theory, systematically erodes relational trust. Psychological safety serves as the pivotal psychological foundation underpinning interactions among employees in the workplace, acting as a bridge within this dynamic. When employees perceive a secure environment, they are more inclined to openly share their perspectives and offer suggestions. When employees who are the targets of gossip face the threat of a breakdown in their psychological safety, they often respond through a dual-channel approach: actively pursuing alternative employment opportunities while concurrently disengaging from organization (Cropanzano and Mitchell, 2005). Conservation of resource theory explains the behavior patterns or coping mechanisms of individuals in the face of stressors (individuals seek protection or access to resources; Hobfoll, 1989, 1988). If the employee has lost resources or is at risk of resource loss in the near future, (s)he experiences stress or threatened. They are then likely to look for resources that are less risky or more favorable to them according to some conditions of the workplace environment (Hobfoll and Freedy, 1993). Specifically, negative workplace gossip (i.e., negative interactions) can threaten employees' psychological safety resources; this increases pressure on employees and can change their attitudes (Bartram et al., 2012)—to retain resources, their intention to leave is likely to increase. Conversely, employees with a relatively satisfactory perception of psychological safety have lower turnover intention (Brohi et al., 2018; Pratt, 1998), and they are even more willing to stay and work harder for the current organization (Eivazzadeh and Nadiri, 2022; Ulusoy et al., 2016). So, this study put forward the second hypothesis:

*Hypothesis 2: Psychological safety functions as the mediating mechanism transmitting negative workplace gossip's influence on kindergarten teachers' turnover intention*.

### Organizational identification as a moderating factor

As defined by Ashforth and Mael (1989), organizational identification refers to employees' emotional stance toward a particular team or organization, encapsulating their feelings of belonging, responsibility, dependence, and organizational recognition. Employees who strongly identify with their organization not only foster greater trust in the workplace environment and colleagues but also exhibit a more profound sense of connection and loyalty to the organization (Kreiner and Ashforth, 2004; Jiang et al., 2024). Organizational identification was identified as a non-negligible variable to predict turnover intention (Niu et al., 2022; Yang and Lu, 2023). When employees' organizational identification grows, it results in a reduction of their turnover intention (Shaikh et al., 2022). Huang and Lin (2017) pointed out that when it comes to ethical issues, organizational identification exerts an indispensable and important function in the dynamic change of employees' psychology and the emergence of turnover intention. Previous studies treating organizational identification as a moderator demonstrated that its high levels among employees effectively mitigated the detrimental effects of limited psychological resources on organizational conduct (Liu et al., 2021; Xiao et al., 2025). Specifically, organizational identification can enhance the level of psychological safety and be transformed into an organizational safety climate (Xia et al., 2025). The concurrent enhancement of organizational identification and psychological safety fosters collaborative interdependence among colleagues, amplifies goal-oriented behavioral engagement, and systematically reduces turnover intention while strengthening organizational retention motives (Boon et al., 2020; Whitman, 2025). Conversely, employees' turnover intention will increase as organizational identification declines (Chang et al., 2021). Thus, the third hypothesis can be reasonably articulated as:

*Hypothesis 3: Organizational identification moderates the association between psychological safety and kindergarten teachers' turnover intention. Psychological safety has a stronger association with turnover intention among kindergarten teachers with a high level of organizational identification than among those with a low level of organizational identification*.

### The present study

**Figure 1:**
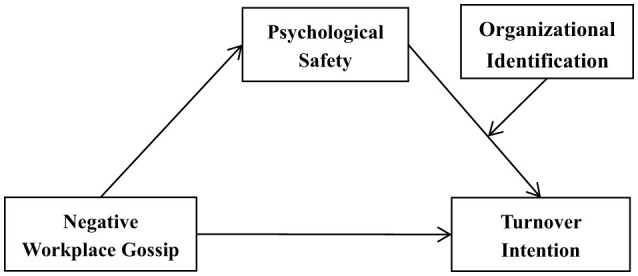
Potential moderated-mediation framework.

Grounded in social exchange theory (Blau, 1964) and conservation of resources theory (Hobfoll, 1989), this investigation advances a potential moderated-mediation framework (Figure 1) to elucidate the dynamic precursors governing turnover intention trajectories within Chinese kindergarten teachers, building upon three theoretically derived propositions. The current study findings can be used as theoretical guidance for kindergarten principals to prevent and reduce negative workplace gossip in the future, and offers suggestions and references for developing intervention measures to manage and control teachers' turnover intention.

## Methods

### Participants and procedure

Data collection was conducted via a questionnaire survey, as this method allows for the systematic quantification of subjective variables among kindergarten teachers through standardized items, providing an empirical foundation for large-scale analysis of multivariate relationships and validation of theoretical models (Dillman et al., 2014). Electronic questionnaires were publicly and randomly distributed to kindergarten teachers in China through Internet (https://www.wjx.cn/) in February 2025. In total, 2,011 kindergarten teachers voluntarily participated in the survey and completed the self-assessment questionnaires. After excluding invalid responses, the data from 1,872 kindergarten teachers from 24 Chinese provinces were selected for inclusion, with a valid response rate of 93.09%. Table 1 outlines the characteristics of the study's sample.

**Table 1:** Sample characteristics (*n* = 1,872).

**Variable**	**Categorical variable coding**	**Categorical variable**	**Frequency (*n*)**	**Percentage (%)**
Gender	1	Male	57	3.04
	2	Female	1,815	96.96
Marital status	1	Unmarried	838	44.76
	2	Married	1,034	55.24
Age	1	18–20	126	6.73
	2	21–30	988	52.78
	3	31–40	466	24.89
	4	41–50	236	12.61
	5	51+	56	2.99
Education level	1	High school diploma	88	4.70
	2	Associate degree	835	44.60
	3	Bachelor's degree	918	49.04
	4	Master's degree	31	1.66

### Measures

#### Perceived negative workplace gossip

Chandra and Robinson (2009) developed the Perceived Negative Workplace Gossip, which was used to measure negative workplace gossip in this study. The scale comprises three items, including “In the past 6 months, others (e.g., coworkers and/or supervisors) communicated damaging information about me in the workplace.” Responses were rated on a five-point Likert scale, with 1 indicating *strongly disagree* and 5 signifying *strongly agree*. Composite mean scores were calculated for each participant, where higher scores reflected greater perceived exposure to negative workplace gossip. The scale's validity in Chinese contexts has been previously established by Cheng et al. (2020) and He and Wei (2022). In the present study, this measure demonstrated excellent internal consistency, achieving a Cronbach's alpha coefficient of 0.96.

#### Intent to Leave Scale

The Intent to Leave Scale developed by Scott et al. (1999) was employed to evaluate employees' turnover intention. This measurement tool is composed of four items, including the example “I would prefer another, more ideal job than the one I now work in.” Respondents' answers to these items were rated on a five-point Likert scale, where 1 represents “*strongly disagree*” and 5 represents “*strongly agree*.” The average scores were computed, and a higher score was indicative of a greater inclination to leave the current job. The Chinese adaptation of this scale has been utilized in the study by Fan et al. (2024). In the present research, the scale exhibited a satisfactory level of internal consistency, with a Cronbach's alpha coefficient of 0.85.

#### Organizational Identification Scale

To measure organizational identification, we employed the six-item Organizational Identification Scale developed by Mael and Ashforth (1992). An example item from this scale is “When I talk about my organization, I usually say ‘we' rather than ‘they'.” Participants were asked to provide their responses to each item on a five-point rating scale, where 1 stands for “*strongly disagree*” and 5 represents “*strongly agree*.” We then computed the mean scores. A higher mean score was associated with a greater degree of organizational identification. Previous research by Teng et al. (2020) demonstrated that the Chinese adaptation of this scale possesses good validity. In the present study, the scale achieved a Cronbach's alpha coefficient of 0.90, indicating high internal consistency.

#### Team Psychological Safety Scale

Psychological safety was evaluated using the Team Psychological Safety Scale adapted by Li and Yan (2007), which draws from the original framework developed by May et al. (2004). This measurement tool comprises five items, including the example: “In my job, I don't have to look over my shoulder all the time.” Participants indicated their agreement with each item on a seven-point Likert scale, where 1 signified very inaccurate and 7 indicated very accurate. To ensure consistency, Items 2, 3, 4, and 5 underwent reverse-scoring during data processing. Composite mean scores were calculated, with higher values reflecting stronger perceptions of psychological safety. In the present investigation, the scale demonstrated a Cronbach's alpha coefficient of 0.76, indicating acceptable internal consistency for the measure.

### Statistical analysis

Statistical analyses were conducted using IBM SPSS Statistics 24.0 and AMOS 29.0. First, common method bias was assessed via Harman's single-factor test and multiple linear regression analysis. Subsequently, the dataset underwent descriptive statistics, analysis of variance (ANOVA), and correlation analysis to characterize variables and examine bivariate associations. Next, confirmatory factor analysis (CFA) of the research model was performed using AMOS 29.0 within a structural equation modeling (SEM) framework, ensuring construct validity and measurement reliability. Finally, the theoretical models were evaluated to test: (1) psychological safety as a mediator in the relationship between negative workplace gossip and kindergarten teachers' turnover intention; and (2) organizational identity plays a moderating role in the second half of this indirect approach. Hayes's (2013) PROCESS macro (version 4.1) was employed, with Model 4 testing the mediation effect through psychological safety and Model 14 examining the moderation effect by organizational identification. Then, 5,000 bias-corrected bootstrapped resamples were used to estimate the 95% confidence interval (CI; statistical significance is indicated if the confidence interval does not contain zero) of the mediating and moderating effects (Preacher and Hayes, 2008). Following Weng et al. (2018), age, gender, marital status, and education were controlled for as they may relate to turnover intention.

## Results

### Latent variable measurement models

To assess common method biases across the four questionnaire scales, Harman's one-factor method was employed. Principal component analysis of all variables was conducted, yielding six components with eigenvalues >1. The first component explained 33.95% of the total variance—below the 40% critical cut-off for severe common method bias (Podsakoff et al., 2003)—indicating no serious contamination from such biases in this study. Multi-collinearity among the four variables was examined using multiple linear regression to calculate variance inflation factor (VIF) values. All VIF scores (ranging from 1.164 to 2.146) remained well under the conventional threshold of 5 (Hair et al., 2011), providing no evidence of problematic multi-collinearity.

This research employed AMOS 29.0 to perform confirmatory factor analyses for comparing four hypothesized structural equation models, as outlined in Table 2. Following widely accepted fit criteria (e.g., CFI > 0.90, RMSEA < 0.08; Ferreira-Valente et al., 2016), a comparative evaluation of the one-factor, two-factor, three-factor, and hypothesized four-factor models revealed that the four-factor structure outperformed alternatives across all fit indices (χ^2^/*df* = 3.338, TLI = 0.986, CFI = 0.990, GFI = 0.980, NFI = 0.986, IFI = 0.990, and RMSEA = 0.035). These results indicate that the four-factor structure more accurately captures the underlying relationships among variables, enhancing both the explanatory power and robustness of the model.

**Table 2:** Comparative fit indices for hypothesized structural equation model configurations.

**Model**	**χ^2^**	**χ^2^*/df***	***TLI***	***CFI***	***GFI***	***NFI***	***IFI***	***RMSEA***
One-factor model Combining NWG, PS, OI, and TI	12110.317	89.706	0.452	0.516	0.454	0.514	0.517	0.218
Two-factor model Combining NWG, PS, and OI	1129.153	11.070	0.938	0.959	0.940	0.955	0.959	0.073
Three-factor model Combining NWG and PS	926.739	8.274	0.955	0.967	0.949	0.963	0.967	0.062
Four-factor model NWG, PS, OI, and TI	374.175	3.338	0.986	0.990	0.980	0.986	0.990	0.035

NWG, Negative workplace gossip; PS, Psychological safety; OI, Organizational identification; TI, Turnover intention.

### Preliminary analyses

Table 3 shows the results of the one-way multivariate analysis of variance (MANOVA). The average score for turnover intention among kindergarten teachers in China was 2.67 ± 1.09 (range from 1 to 5), which is lower than the theoretical median score (3.00) and shows that, in general, the teaching staff of kindergartens in China have high stability. However, 35.15% of kindergarten teachers had an average turnover intention higher than the theoretical median score (3.00), which means that these kindergarten teachers are at higher risk of leaving their jobs in the future. The results of the analysis of variance showed that the scores for turnover intention among kindergarten teachers were significantly different based on demographic characteristics such as age (*p* < 0.001), gender (*p* < 0.05), marital status (*p* < 0.001), and education level (*p* < 0.001). Specifically, higher turnover intention scores among younger teachers relative to older teachers, male teachers compared to female teachers, and unmarried teachers vs. married teachers, and teachers with a higher level of education had a stronger turnover intention.

**Table 3:** One-way multivariate analysis of variance results (*n* = 1,872).

**Subject variables**	**Turnover intention**		**Negative workplace gossip**		**Psychological safety**		**Organizational identification**	
	***M (SD)***	***F/t***	***M (SD)***	***F/t***	***M (SD)***	***F/t***	***M (SD)***	***F/t***
Age	2.67 (1.09)	91.89^***^	1.98 (1.10)	8.23^***^	5.14 (1.30)	4.08^**^	4.03 (0.81)	48.77^***^
**Gender**								
Male	2.97 (1.10)	2.14^*^	2.48 (1.22)	3.50^***^	4.78 (1.31)	−2.14^*^	3.68 (0.89)	−3.28^**^
Female	2.66 (1.09)		1.96 (1.09)		5.15 (1.29)		4.04 (0.81)	
**Marital status**								
Unmarried	3.06 (1.02)	220.13^***^	2.08 (1.10)	13.32^***^	5.04 (1.30)	9.95^**^	3.78 (0.82)	153.87^***^
Married	2.35 (1.05)		1.90 (1.09)		5.23 (1.28)		4.23 (0.75)	
**Educational level**								
High school diploma	2.27 (1.10)	11.21^***^	1.78 (1.15)	2.06	5.10 (1.31)	0.85	4.20 (0.76)	3.85^**^
Associate degree	2.81 (1.09)		1.94 (1.10)		5.10 (1.31)		3.97 (0.85)	
Bachelor's degree	2.57 (1.08)		2.03 (1.09)		5.19 (1.28)		4.07 (0.79)	
Master's degree	2.83 (1.04)		2.05 (1.04)		5.08 (1.19)		3.93 (0.72)	

M, Mean; SD, Standard Deviation. ^*^p < 0.05, ^**^p < 0.01, and ^***^p < 0.001.

The average score of negative workplace gossip was 1.98 ± 1.10, the median value of negative workplace gossip was 2.00, and the average score of 88.51% of kindergarten teachers was lower than the theoretical median value (3.00), demonstrating that the negative workplace gossip in kindergartens is not serious. Here, too, the scores varied significantly based on the demographic characteristics of age (*p* < 0.001), gender (*p* < 0.001), and marital status (*p* < 0.001), but there was no statistical significance based on educational level (*p* > 0.05). Specifically, higher negative workplace gossip scores among younger teachers relative to older teachers, male teachers compared to female teachers, and unmarried teachers vs. married teachers.

The average psychological safety score among kindergarten teachers was 5.14 ± 1.30 (range from 1 to 7), and the median score was 5.40; 77.62% of kindergarten teachers had a perceived psychological safety average score higher than the theoretical median (4.00), which indicates that the psychological climate of these kindergartens is relatively relaxed and free. The psychological safety scores differed significantly by age (*p* < 0.01), gender (*p* < 0.05), and marital status (*p* < 0.01), and there was no significant difference based on educational level (*p* > 0.05). Specifically, lower psychological safety scores among younger teachers relative to older teachers, male teachers compared to female teachers, and unmarried teachers vs. married teachers.

The average organizational identification score was 4.03 ± 0.81. The results of the analysis of variance indicated significant differences in the organizational identification scores based on age (*p* < 0.001), gender (*p* < 0.01), marital status (*p* < 0.001), and educational level (*p* < 0.01). Specifically, younger teachers had lower organizational identification scores than older teachers, male teachers had lower scores compared to female teachers, and unmarried teachers had lower scores vs. married teachers, and teachers with a higher level of education had a lower organizational identification.

As presented in Table 4, the CFA results yielded composite reliability (CR) values of 0.956 (negative workplace gossip), 0.829 (psychological safety), 0.902 (Organizational identification), and 0.852 (turnover intention); all CR values were higher than 0.7 and so could be considered acceptable and good (Ferreira-Valente et al., 2016). The values for the average variance extracted (AVE) were 0.884 (negative workplace gossip), 0.559 (psychological safety), 0.601 (organizational identification), and 0.616 (turnover intention); all AVE values were higher than 0.5, which suggests adequate convergent validity (Ferreira-Valente et al., 2016). To sum up, the measurement items can effectively reflect their respective constructs. In addition, the test of discriminant validity further confirms that the square roots of AVE for each construct are greater than their correlation coefficients with other constructs (see Table 3), meeting the Fornell and Larcker (1981) criterion. It follows that the variables here met the criteria for both convergent and discriminant validity.

**Table 4:** The correlation results between the measured variables (*n* = 1,872).

**Variables**	**α**	**CR**	**AVE**	***M***	***SD***	**1**	**2**	**3**	**4**	**5**	**6**	**7**	**8**
1. Age				2.52	0.90	1							
2. Gender				1.97	0.17	−0.01	1						
3. Marital status				1.55	0.50	0.63^***^	0.01	1					
4. Education level				2.48	0.61	0.01	−0.09^***^	0.09^***^	1				
5. Negative workplace gossip	0.96	0.956	0.879	1.98	1.10	−0.11^***^	−0.08^***^	−0.09^***^	0.06^**^	(**0.938**)			
6. Psychological safety	0.76	0.829	0.552	5.14	1.30	0.08^***^	0.05^*^	0.08^**^	0.03	−0.72^***^	(**0.743**)		
7. Organizational identification	0.90	0.902	0.605	4.03	0.81	0.29^***^	0.08^**^	0.28^***^	0.02	−0.11^***^	0.14^***^	(**0.778**)	
8. Turnover intention	0.85	0.884	0.668	2.67	1.09	−0.39^***^	−0.05^*^	−0.33^***^	−0.03	0.43^***^	−0.41^***^	−0.37^***^	(**0.817**)

M, Mean; SD, Standard Deviation. ^*^p < 0.05, ^**^p < 0.01, and ^***^p < 0.001. The bold values on the diagonal are the arithmetic square roots of the average variance extraction values.

Table 4 summarizes variable descriptives and bivariate correlations. As hypothesized, negative workplace gossip showed significant negative associations with psychological safety (*r* = −0.72, *p* < 0.001) and organizational identification (*r* = −0.11, *p* < 0.001), but a positive association with turnover intention (*r* = 0.43, *p* < 0.001). Psychological safety and organizational identification were positively correlated (*r* = 0.14, *p* < 0.001), while both variables demonstrated inverse relationships with turnover intention (*r* = −0.41 and *r* = −0.37, respectively; *p* < 0.001 for all).

### Testing the mediation model

Model 4 of Hayes's (2013) PROCESS macro was utilized to assess the proposed mediation model. After controlling for demographic variables such as age, gender, marital status, and educational level, workplace gossip had a positive correlation with turnover intention when there was no mediator (β = 0.38, *p* < 0.001). Table 5 indicates that with psychological safety taken into account, negative workplace gossip was inversely related to psychological safety (β = −0.85, *p* < 0.001). In turn, psychological safety was found to be negatively linked to turnover intention (β = −0.18, *p* < 0.001). The association between negative workplace gossip and turnover intention was significant (β = 0.23, *p* < 0.001). Furthermore, as presented in Table 6, both the indirect effect of psychological safety (95% CI [0.11, 0.20]) and the direct effect of negative workplace gossip on turnover intention (95% CI [0.18, 0.28]) reached statistical significance, with confidence intervals excluding zero. The mediation effect made up 39.47% of the total effect. The results indicate that psychological safety partially mediates the relationship between negative workplace gossip and turnover intention among Chinese kindergarten teachers.

**Table 5:** Psychological safety's mediating effect on negative workplace gossip—turnover intention relationship.

**Predictors**	**Psychological safety**			**Turnover intention**		
	β	***SE***	***95% CI***	β	***SE***	***95% CI***
Constant	6.48^***^	0.28	[5.94, 7.02]	4.82^***^	0.31	[4.20, 5.43]
Age	−0.01	0.03	[−0.07, 0.05]	−0.33^***^	0.03	[−0.39, −0.27]
Gender	−0.02	0.12	[−0.26, 0.22]	−0.16	0.12	[−0.40, 0.07]
Marital status	0.02	0.05	[−0.09, 0.13]	−0.25^***^	0.05	[−0.36, −0.14]
Education level	0.15^***^	0.03	[0.08, 0.21]	−0.05	0.03	[−0.12, 0.01]
Negative workplace gossip	−0.85^***^	0.02	[−0.89, −0.81]	0.23^***^	0.03	[0.18, 0.28]
Psychological safety				−0.18^***^	0.02	[−0.23, −0.14]
*R^2^*	0.51			0.33		
*F*	399.16^***^			154.39^***^		

The total number of bootstrap samples was 5,000. ^*^p < 0.05, ^**^p < 0.01, and ^***^p < 0.001.

**Table 6:** Total, direct, and indirect effects.

**Effect type**	**Estimated effect**	***SE***	***95% CI***	**Ratio to the total effect**
Total effect	0.38	0.02	[0.35, 0.42]	
Direct effect	0.23	0.03	[0.18, 0.28]	60.53%
Indirect effect	0.15	0.02	[0.11, 0.20]	39.47%

The total number of bootstrap samples was 5,000.

### Testing the moderated mediation model

**Figure 2:**
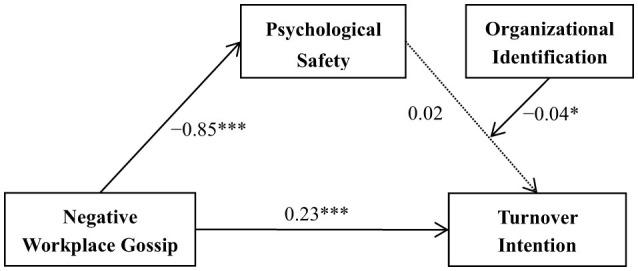
Moderated mediation pathways of negative workplace gossip, psychological safety, organizational identification, turnover intention. *p < 0.05 and ***p < 0.001.

Model 14 of Hayes's (2013) PROCESS macro was utilized to assess the proposed moderated—mediation model, in which psychological safety functioned as the mediator and organizational identification as the moderator, with age, marital status, gender, and educational level as covariates. As shown in Table 7, the interaction effect of organizational identification and psychological safety had a significant effect on turnover intention among Chinese kindergarten teachers (β = −0.04, *p* < 0.05), and organizational identification as a moderator was also confirmed (*95% CI* = [−0.08, −0.01]). This finding indicates that organizational identification moderates the association between psychological safety and turnover intention. Figure 2 outlines the moderated mediation pathway with path weights included.

**Table 7:** Moderated mediation analyses.

**Predictors**	**Psychological safety**			**Turnover intention**		
	β	***SE***	***95% CI***	β	***SE***	***95% CI***
Constant	6.48^***^	0.28	[5.94, 7.02]	4.59^***^	0.49	[3.64, 5.54]
Age	−0.01	0.03	[−0.07, 0.05]	−0.28^***^	0.03	[−0.33, −0.22]
Gender	−0.02	0.12	[−0.26, 0.22]	−0.06	0.12	[−0.29,0.18]
Marital status	0.02	0.05	[−0.08, 0.13]	−0.18^***^	0.05	[−0.28, −0.07]
Educational level	0.15^***^	0.03	[0.08, 0.21]	−0.05	0.03	[−0.11, 0.02]
Negative workplace gossip	−0.85^***^	0.02	[−0.89, −0.81]	0.23^***^	0.03	[0.18, 0.28]
Psychological safety				0.02	0.08	[−0.14, 0.17]
Organizational identification				−0.09	0.10	[−0.28, 0.10]
Psychological safety × Organizational identification				−0.04^*^	0.02	[−0.08, −0.01]
*R^2^*	0.51			0.38		
*F*	399.16^***^			142.53^***^		

The total number of bootstrap samples was 5,000. ^*^p < 0.05 and ^***^p < 0.001.

**Figure 3:**
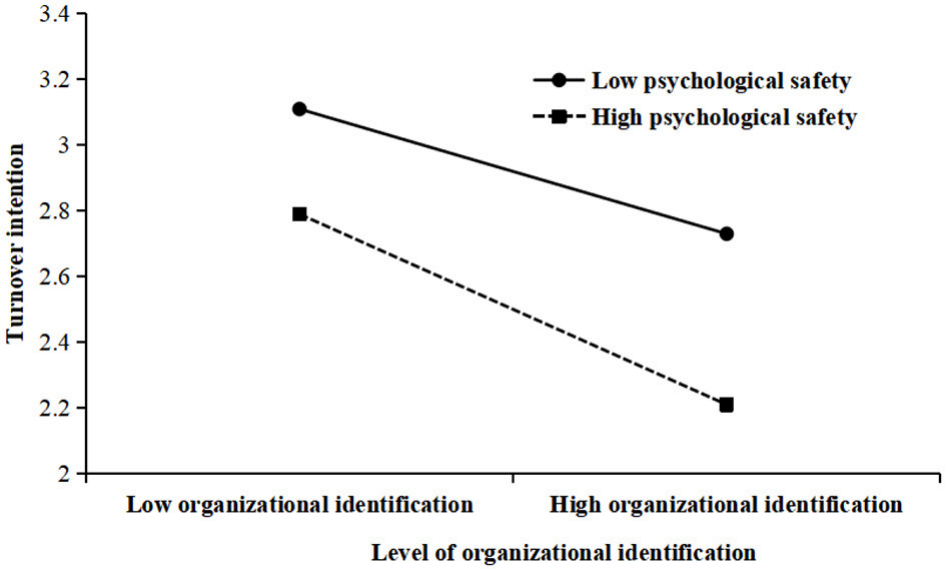
Relationship between psychological safety with turnover intention.

To ascertain the features of the moderating impacts exerted by organizational identification, a simple slope analysis was conducted. The association between psychological safety with kindergarten teachers' turnover intention at two levels of organizational identification (low level, M – 1 SD; high level, M + 1 SD) was plotted. As shown in Figure 3, the simple slope tests suggested that for kindergarten teachers with low organizational identification, psychological safety was negatively associated with turnover intention (simple slope = −0.12, *p* < 0.001), while the effect was much weaker for kindergarten teachers with high organizational identification (simple slope = −0.19, *p* < 0.001).

## Discussion

The present study sought to clarify the mediating and moderating mechanisms in the association between negative workplace gossip and turnover intention among Chinese kindergarten teachers using a moderated mediation model. As expected, the findings of the present study indicated that psychological safety partially mediated the association between negative workplace gossip and turnover intention. Organizational identification moderated the association between psychological safety and turnover intention.

### Discussion of findings regarding the hypotheses

Empirical data confirmed that negative workplace gossip exhibited a direct positive relationship with turnover intention, which verifies Hypothesis 1 and reinforces conclusions drawn in earlier scholarly work (He and Wei, 2022; Xie et al., 2019) and social exchange theory. The mechanism of individual interaction can be found in social exchange theory, where employees distinguish the risks and benefits of social interaction, including with colleagues, through cost-benefit analysis. Unfavorable conditions in the work environment can lead employees to seek more favorable situations (Cropanzano and Mitchell, 2005). Negative workplace gossip, as an abusive and indirect abnormal interaction pattern in the social field, often threatens the normal work order of employees due to its uncertain negative impact; to remove this threat, turnover intention may increase. The empirical data also revealed that psychological safety partially mediated the relationship between negative workplace gossip and kindergarten teachers' turnover intention, which verifies Hypothesis 2. This result is consistent with conservation of resource theory, when employees are threatened by negative information about themselves, their perception of resource loss increases (Naeem et al., 2019), and their psychological safety—already limited—becomes further eroded, prompting them to actively seek ways to preserve and recover their resources (Hong et al., 2024). Negative workplace gossip leads to a decrease in teachers' perception of psychological safety, and teachers may increase their turnover intention in order to seek or retain resources related to psychological safety.

This study also found that organizational identification moderated the association between psychological safety and turnover intention, thus supporting Hypothesis 3. More specifically, organizational identification enhanced the influence of psychological safety on turnover intention. According to Huang and Lin (2017), when ethical issues arise within an organization, organizational identification will play a crucial driving role in the psychological processes of employees and the evolution of their turnover intention. This study confirmed this mechanism. When kindergarten teachers are harmed by negative workplace gossip, they may perceive the organization as filled with serious psychological safety threats. However, if they strongly identify with the kindergarten, this can help them better cope with their ego depletion and emotional exhaustion (Liu et al., 2020; Ullah et al., 2021). Johnson and Avolio found organizational identification to be positively correlated with psychological safety (Johnson and Avolio, 2019), and organizational identification can positively moderate psychological safety (Chen et al., 2019). If kindergarten teachers identify with an organization, the resulting enhancement in the sense of belonging supports the integration of the teacher self-concept into the perception of the organization—that is, the more teachers identify with the kindergarten they work in, the more likely they are to stay on (Chang et al., 2021).

### Theoretical contributions

First, this study expands existing gossip dissemination theories by evolving the understanding of negative workplace gossip from unidirectional harm to bidirectional dynamics. Grounded in the bystander effect (Darley and Latané, 1968; Latané and Darley, 1968), this study constructs a “victim-bystander” dual-role interaction model, highlighting that the same early childhood teacher may concurrently assume both roles across different contexts (e.g., as the target of current gossip and a bystander to past gossip), with their turnover intention emerging as the cumulative outcome of multiple experiences.

Second, by adopting a human resource management perspective, this work enriches the understanding of social exchange theory. Researchers in this area unanimously believe that social exchange involves a series of interactions that generate obligations (Emerson, 1976), which are commonly regarded as interdependent and depend on the behavior of another person (Blau, 1964). Cropanzano and Mitchell (2005) pointed out that although the framework of social exchange theory is useful, its structure has not yet been completely determined. This study may provide support for addressing this point. Negative workplace gossip is a one-way, indirect exchange of information: There is no obligation to interact between the giver of the gossip and the target of the gossip. However, we argue that negative workplace gossip and turnover intention are two interdependent variables in a social exchange. Specifically, negative workplace gossip positively affects employees' turnover intention, and the increase in turnover intention in turn reminds kindergarten principals to eliminate or control the frequency of negative workplace gossip and the scope and degree of its impact to ensure the stability of the organization or team.

Third, this study has expanded the paths for resource protection and acquisition, and enriched the connotation of the conservation of resource theory. The core perspective of this theory is that human beings have an inherent motivation to protect existing resources and acquire new resources (Halbesleben et al., 2014). Resources here include objects, states, conditions, and other things that people hold dear (Hobfoll, 1988). In practical work scenarios, this theory has specific manifestations. For example, when employees are affected by negative workplace gossip, they may feel that their control over important resources such as privacy, reputation, and emotions is diminishing, which in turn leads to a decrease in their psychological security. Based on the principles of resource conservation and timely loss prevention, when employees' psychological security resources are damaged, their turnover intention may increase significantly. Certainly, employees will not only actively protect existing resources but also, under the influence of other factors, take the initiative to seek and acquire new resources. When employees possess abundant psychological capital, they are more inclined to continue serving the organization they belong to Abdou et al. (2025). This study has found that by enhancing the organizational identification of kindergarten teachers who have been or are currently being affected by negative workplace gossip, the loss of psychological security resources can be compensated for, and their intention to leave their jobs can be effectively reduced.

### Implications for practice

Although this study focused on a specific sample, the findings may have broader implications. First, the survey results shed light on the current situation of turnover intention among kindergarten teachers in China, which was at a moderate to lower level (2.67 ± 1.09, ranging from 1 to 5), consistent with the research results of He and Wei (2022; 2.45 ± 0.85, ranging from 1 to 5), but is higher than the previously published average scores for and Spain (1.40 ± 1.08, ranging from 1 to 5; Mérida-López et al., 2020) in the same period. Although the average score of kindergarten teachers' turnover intention in China was slightly higher than that in other countries and regions of the world, it was lower than the theoretical median score (3.00), which indicates that the stability of the team of kindergarten teachers is under low potential threat from resignation. The findings also showed, however, that 35.15% of kindergarten teachers were at risk of leaving their jobs in the future. Kindergarten principals should therefore pay attention to the factors influencing turnover intention. We found that younger, unmarried, or male teachers were more likely to be the targets of negative workplace gossip; simultaneously, their levels of psychological safety and organizational identification were significantly lower. These groups therefore tended to have higher turnover intention. Kindergarten principals should therefore pay more attention to these teacher groups from a human resource management perspective.

Second, Although the phenomenon of negative workplace gossip in kindergartens is not prominent or serious, kindergarten principals still need to pay attention to it and take measures to prevent and deal with it, as this may increase the degree of loss of teachers' psychological safety and trigger stronger turnover intention. As highlighted by some researchers, negative workplace gossip constitutes a toxic manifestation of organizational culture that systematically enables psychological abuse through covert relational aggression, which is constantly carried out with vicious intent and can lead to low employee morale, harming individuals and organizations (Tian et al., 2018). Workplace gossip should be eliminated. Kindergarten principals need to simultaneously address the “direct harm to gossip targets” and the “indirect impact on bystanders.” For example, establishing “bystander support mechanisms”—such as encouraging positive communication and setting up anonymous reporting channels—can disrupt the transmission chain of “silence-harm-identity erosion” (He and Wei, 2022), thereby reducing the risk of teacher turnover caused by dual-role stress.

Third, these findings offer some hope for kindergartens. Organizational identification can alleviate the effect of poor perceived psychological safety on turnover intention. Kindergarten principals should therefore pay more attention to negative gossip behavior and the status of interpersonal relationships among colleagues in the organization. They should also take practical and effective measures to enhance organizational identification and psychological safety of kindergarten teachers to reduce or eliminate turnover intention.

### Limitations and future directions

Despite efforts to avoid design flaws, this study still has some limitations. Firstly, The synchronic nature of cross-sectional data precludes causal-temporal mapping, requiring diachronic designs to delineate variables' temporal ordering. Second, the cultural specificity of the Chinese sample (96.96% female) constrains the ecological validity of findings, necessitating cross-cultural replications with gender-balanced cohorts in future research. Third, while prior research has established negative workplace gossip as an antecedent of turnover intention, this relationship is still questionable because negative workplace gossip does not necessarily lead to negative results for the target. Tan et al. (2021) confirmed through three rounds of research that negative workplace gossip could increase performance pressure and lead to improved job performance. Researchers therefore should change the stereotype of negative workplace gossip in future studies and focus on its positive value.

## Conclusions

This study identifies psychological safety as a mediator and organizational identification as a moderator in the relationship between negative workplace gossip and turnover intention among kindergarten teachers. Negative gossip reduces perceived psychological safety (the sense of security in expressing oneself), which increases turnover intention. However, strong organizational identification weakens this effect, showing that a sense of belonging to the organization buffers the impact of low psychological safety on teachers' desire to leave. These findings extend conservation of resource theory by demonstrating how relational (psychological safety) and organizational (identification) resources interact to influence turnover. Practically, kindergarten principals should address gossip by promoting psychological safety and fostering organizational identification to retain teachers in this high-demand profession.

## Data Availability

The raw data supporting the conclusions of this article will be made available by the authors, without undue reservation.
